# Code Stroke Alert: Focus on Emergency Department Time Targets and Impact on Door-to-Needle Time across Day and Night Shifts

**DOI:** 10.3390/jpm14060596

**Published:** 2024-06-02

**Authors:** Florina Buleu, Daian Popa, Carmen Williams, Anca Tudor, Dumitru Sutoi, Cosmin Trebuian, Covasala Constantin Ioan, Aida Iancu, Gabriel Cozma, Ana-Maria Marin, Ana-Maria Pah, Ion Petre, Ovidiu Alexandru Mederle

**Affiliations:** 1 Department of Cardiology, “Victor Babes” University of Medicine and Pharmacy, E. Murgu Square No. 2, 300041 Timisoara, Romania; florina.buleu@umft.ro (F.B.); anamaria.pah@umft.ro (A.-M.P.); 2 Department of Surgery, Emergency Discipline, “Victor Babes” University of Medicine and Pharmacy, 300041 Timisoara, Romania; dumitru.sutoi@umft.ro (D.S.); trebuian.cosmin@umft.ro (C.T.); mederle.ovidiu@umft.ro (O.A.M.); 3 Doctoral School, Faculty of General Medicine, “Victor Babes” University of Medicine and Pharmacy Timisoara, 300041 Timisoara, Romania; petre.ion@umft.ro; 4 Emergency Municipal Clinical Hospital, 300254 Timisoara, Romania; drcarmen.williams@yahoo.com (C.W.); gabriel.cozma@umft.ro (G.C.); 5 Department of Functional Sciences, “Victor Babes” University of Medicine and Pharmacy, E. Murgu Square No. 2, 300041 Timisoara, Romania; atudor@umft.ro; 6 Emergency Clinical Hospital, 310003 Arad, Romania; covasala.constantin@gmail.com; 7 Department of Radiology, “Victor Babes” University of Medicine and Pharmacy, E. Murgu Square No. 2, 300041 Timisoara, Romania; aida.parvu@umft.ro; 8 Department of Surgical Semiology, Faculty of Medicine, “Victor Babes” University of Medicine and Pharmacy Timisoara, 300041 Timişoara, Romania; 9 Department of Parasitology and Parasitic Diseases, University of Life Sciences “King Mihai I” from Timisoara, Calea Aradului 119, 300645 Timisoara, Romania; anamaria.marin@usvt.ro

**Keywords:** emergency department, ED time targets, door-to-needle time, acute ischemic stroke, rt-PA

## Abstract

Background and objectives: To minimize stroke-related deaths and maximize the likelihood of cerebral reperfusion, medical professionals developed the “code stroke” emergency protocol, which allows for the prompt evaluation of patients with acute ischemic stroke symptoms in pre-hospital care and the emergency department (ED). This research will outline our experience in implementing the stroke code protocol for acute ischemic stroke patients and its impact on door-to-needle time (DTN) in the ED. Methods: Our study included patients with a “code stroke alert” upon arrival at the emergency department. The final sample of this study consisted of 258 patients eligible for intravenous (IV) thrombolysis with an onset-to-door time < 4.5 h. ED admissions were categorized into two distinct groups: “day shift” (from 8 a.m. to 8 p.m.) (n = 178) and “night shift” (from 8 p.m. to 8 a.m.) (n = 80) groups. Results: An analysis of ED time targets showed an increased median during the day shift for onset-to-ED door time of 310 min (IQR, 190–340 min), for door-to-physician (emergency medicine doctor) time of 5 min (IQR, 3–9 min), for door-to-physician (emergency medicine doctor) time of 5 min (IQR, 3–9 min), and for door-to-physician (neurologist) time of 7 min (IQR, 5–10 min), also during the day shift. During the night shift, an increased median was found for door-to-CT time of 21 min (IQR, 16.75–23 min), for door-to-CT results of 40 min (IQR, 38–43 min), and for door-to-needle time of 57.5 min (IQR, 46.25–60 min). Astonishingly, only 17.83% (n = 46) of these patients received intravenous thrombolysis, and the proportion of patients with thrombolysis was significantly higher during the night shift (*p* = 0.044). A logistic regression analysis considering the door-to-needle time (minutes) as the dependent variable demonstrated that onset-to-ED time (*p* < 0.001) and door-to-physician (emergency medicine physicians) time (*p* = 0.021) are predictors for performing thrombolysis in our study. Conclusions: This study identified higher door-to-CT and door-to-emergency medicine physician times associated with an increased DTN, highlighting further opportunities to improve acute stroke care in the emergency department. Further, door-to-CT and door-to-CT results showed statistically significant increases during the night shift.

## 1. Introduction

The incidence of acute ischemic stroke (AIS) is similar to the incidence of acute coronary syndromes. Thus, acute ischemic syndromes are responsible for the majority of cardiovascular-related deaths and, therefore, overall mortality in most countries [1,2]. AIS represents the second leading cause and a significant reason for disability and related costs [3]. According to stroke statistical reports from 2015, Romania emerged as the European country with the highest incidences of new strokes and stroke-related fatalities [4]. 

To increase and optimize the chances of cerebral reperfusion to reduce stroke-related fatalities, the “code stroke” was created, which is an emergency protocol for the immediate assessment of patients with suspected cerebrovascular events [5,6]. This protocol aims to increase the administration of currently available reperfusion therapies for ischemic stroke. However, only a small percentage of stroke patients received intravenous thrombolysis in the first 4.5 h after symptom onset. The primary reason for the low rate of administration is either the delayed arrival of patients at the emergency department [7,8] or the absence of a dedicated stroke team/unit in most hospitals [9]. 

Although this information is available, there needs to be more awareness and implementation of it among medical personnel when it comes to meeting time targets for ED admissions of AIS patients in hospitals without specialized stroke teams or units. Therefore, it is crucial to explore ways to increase the rate of rt-PA administration, especially considering that in Romania, the Romanian Neurology Society board introduced the National Program of Priority Actions in the Interventional Treatment of Patients with Acute Cerebral Vascular Accident (PA-CVA) document in 2018 [5], report that the thrombolysis rate is less than 10% (around 5.4%), with a notable increase in the last five years from 0.8% [9]. 

For this reason, this study aims to analyze ED time targets and their impact on the DTN time in the county’s largest hospital, which has a specialist team ready to respond to a “code stroke alert”.

## 2. Methods 

### 2.1. Study Population and Inclusion and Exclusion Criteria 

We conducted an observational study on patients referred to the Emergency County Clinical Hospital in Arad, Romania, between 1 January 2020 and 31 December 2023 with a “Code Stroke Alert”. This hospital has an annual influx of over 70,000 patients, is the largest hospital in the county, and coordinates residency programs in neurology and emergency medicine. 

Our acute stroke protocol begins with emergency medical services (EMS) personnel identifying a patient with a “code stroke alert” transported by ambulance or the identification of this patient by the triage assistant/emergency physician upon their admission to the ED. Our prehospital acute stroke screening protocol focuses on dispatchers, and paramedics must be able to diagnose stroke using simple tools such as the FAST (Face, Arms, Speech, and Time) indicators, baseline functional status, and current anticoagulants [5]. Immediately after the patient’s admission to the ED, a “code stroke alert” is announced, and the on-duty neurologist examines the patient together with the emergency medicine physician. During this study, our institution had 24/7 coverage by a team consisting of a neurologist, an emergency medicine physician, a radiologist, and an emergency medicine nurse. Moreover, this team could mobilize before the patient’s arrival when the pre-hospital notification was received. The patient is transported to a designated “stroke bed” on arrival. The thrombolytic dose is calculated based on the patient’s weight and prepared if the patient qualifies for thrombosis. The patient’s blood is obtained for laboratory analysis upon admission to the ED, and an intravenous administration of thrombolytics is performed in the ED if the patient meets the criteria for thrombolysis.

We analyzed consecutive patients who had complete medical records (both electronic and paper) and who were labeled with a “code stroke alert” in the emergency department. Of the initial sample of 345 patients, only 258 patients met the criteria for inclusion and exclusion in our study (not eligibility criteria for IV thrombolysis). According to the local protocol, intravenous thrombolysis with rt-PA was initiated within 4.5 h from the onset of the first symptoms of stroke. Patients below 18 years old, those with an initial diagnosis of intracerebral hemorrhage or brain tumor, and those who arrived at the emergency department more than 4.5 h from the onset of stroke symptoms were excluded from our analysis. 

Upon admission, the medical staff recorded the precise moment at which stroke symptoms first appeared, as well as the mode and time of the patient’s arrival at the ED. The patients themselves or their family members provided information on when the initial stroke-related symptoms were, which was noted as the “onset time”. For individuals who experienced symptoms while sleeping, symptom onset was determined as the last period when they were stroke-symptom-free. Ten patients with neurological deficits upon awakening from sleep were considered to have had wake-up strokes. Since it was impossible to assess the exact time of deficit onset, according to our national protocol, these patients were not eligible for IV thrombolysis [5]. The arrival time at the ED was defined as when the patient’s registration was completed at the ED triage office. The onset-to-needle time was calculated as the interval between symptom onset and when IV thrombolysis was performed. All time measurements were expressed in minutes.

Our national protocol [5] recommends the following time targets for stroke management:

- Onset-to-needle time ≤ 4.5 h;
- Door-to-physician time ≤ 10 min (an initial evaluation by both emergency medicine physician and neurologist that includes the time last known to be well, eligibility for IV thrombolysis, and the evaluation of stroke severity);
- Door-to-CT time ≤ 25 min;
- Door-to-CT results ≤ 45 min;
- Door-to-needle time ≤ 60 min.

To accurately establish the patients’ arrival time in the emergency department, we utilized the initial registration time at the ED triage office. This allowed us to categorize ED admissions into two distinct groups: a “day shift” (from 8 a.m. to 8 p.m.) and “night shift” (from 8 p.m. to 8 a.m.). We categorized the mode of arrival—whether it was through EMS or if the patient walked or drove in. We chose these two factors (arrival mode and type of shift) because they are within our realm of influence and have the potential to be modified.

Moreover, by dividing the final sample into two subgroups consisting of 178 patients during the day shift and 80 patients during the night shift, we aimed to analyze the impact of various factors and the management time for acute stroke in the ED on the rate of intravenous rt-PA administration. 

**Figure 1:**
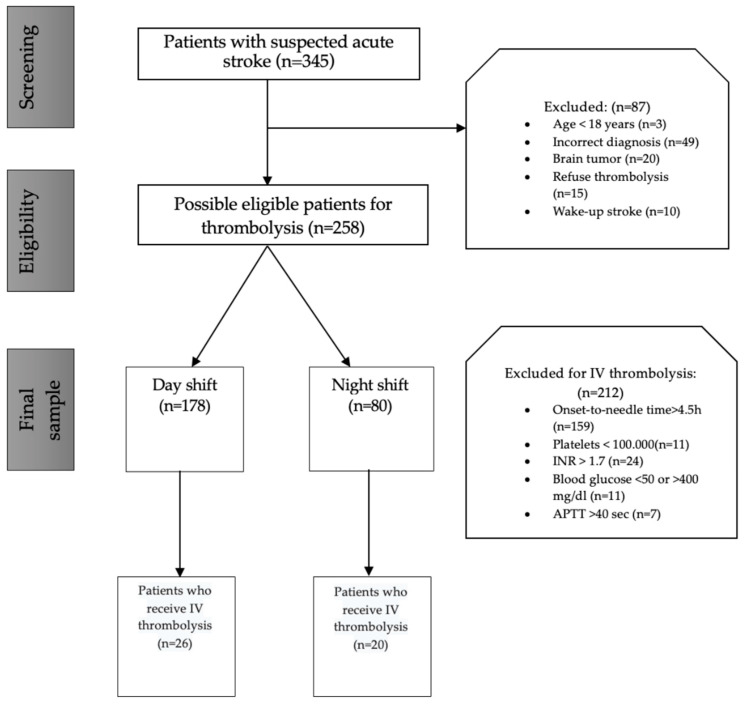
Study flowchart.

As shown in the study flowchart represented in Figure 1, a total of 345 patients with “code stroke alert” and a symptom onset time of less than 4.5 h were screened in the ED for eligibility for intravenous reperfusion therapy. 

Only patients who satisfied the inclusion and exclusion criteria (n = 258) were selected in the final sample of this study. Of these patients, 26 received IV thrombolysis during the day shift; only 20 received IV thrombolysis during the night shift. 

### 2.2. Evaluation of Stroke 

Immediately following admission to the emergency department, all patients undergo a comprehensive medical evaluation which includes a cerebral computed tomography (CT) scan with or without contrast, as well as a complete blood count, international normalized ratio (INR), and prothrombin time, partial prothrombin time, blood glucose, and electrolyte tests. It is important to note that patients without medical data were not included in this evaluation. In conjunction with the emergency medicine physician and neurologist, the radiologist collaboratively assessed the stroke’s subtype, severity, and location based on the brain imaging and clinical examination results according to the stroke definition established by The World Health Organization in 1970, which is still used today [10]. 

The neurologist on duty assessed neurological deficits and stroke severity in the first clinical evaluations of patients in the ED and categorized them using the National Institutes of Health Stroke Scale (NIHSS) at the following intervals: at admission, 1 h, 2 h, and 24 h [11]. 

### 2.3. Ethics

This study was conducted following the Declaration of Helsinki, and the protocol was approved by the Ethics Committee of the Arad County Emergency Clinical Hospital (no. 11687/27 March 2024). The collected data were identified before a statistical analysis was performed. All patients signed the informed consent.

### 2.4. Statistical Analysis 

Mean and standard deviation or median and interquartile range (IQR) values were used to present continuous variables, while frequencies and percentages were used for categorical variables. The distribution of continuous variables was assessed using the Shapiro–Wilk test. Unpaired t-tests or Mann–Whitney U and Chi-square tests were employed to compare the characteristics of patients who received thrombolysis and those who did not. Several multivariate logistic regression models were used to determine the independent factors associated with the administration of thrombolysis. For the sample size calculation, we conducted a power analysis test using the GPower3.1 application for the t-test family—the Wilcoxon–Mann–Whitney test (two groups) with two tails, a normal parent distribution and 95% power, taking 0.05 as the level of significance and 0.5 as the effect size. 

The results were presented as odds ratios with 95% confidence intervals. A *p*-value of less than 0.05 was considered statistically significant. Data analysis was conducted using JASP v0.18.3 (a free and open-source program for statistical analysis supported by the University of Amsterdam).

## 3. Results

### 3.1. Baseline Characteristics of Patients Who Arrived at the Emergency Department

The final sample included 258 patients eligible for IV thrombolysis who were admitted to the ED with a stroke alert. The following results were obtained after dividing them into two subgroups according to their arrival time: those admitted during the day shift (n = 178) and the night shift (n = 80).

**Figure 2:**
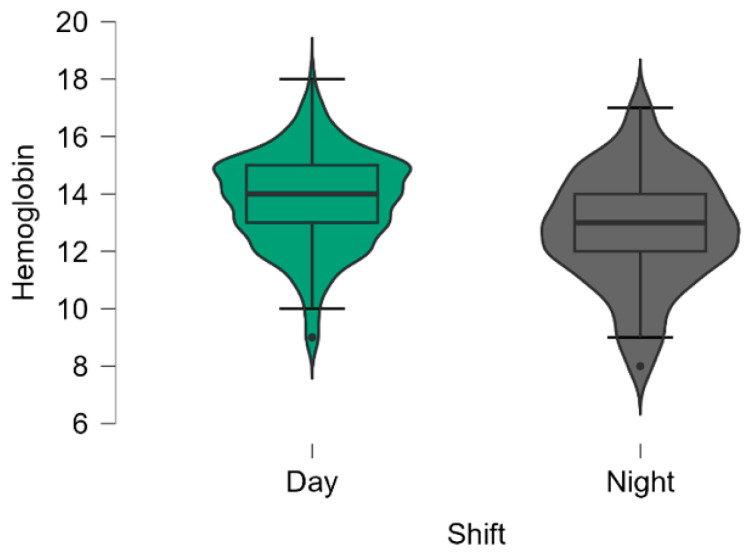
Violin plots of the values of the hemoglobin (mg/dL) between the two types of ED shift (*p* < 0.001). The boxplots inside the violin represent the median and interquartile ranges.

**Figure 3:**
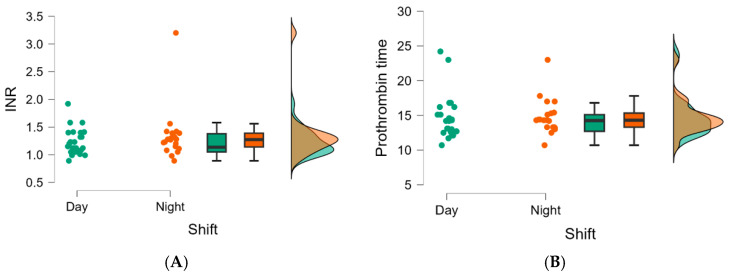
(**A**) Raincloud plots of INR values between the two types of ED shift (*p* = 0.039). (**B**) Raincloud plots of the values of prothrombin time (seconds) between the two types of ED shift (*p* < 0.001).

Table 1 shows the patients’ demographic and clinical characteristics according to the shift type. There were statistically significant differences, with changed values observed only in terms of lower patient height values in the day shift group (*p* = 0.004), lower hemoglobin values in the day shift group (*p* < 0.001), values of increased INR in the night shift group (*p* = 0.039), and a higher prothrombin time in the night shift group (*p* < 0.001). (Table 1, Figure 2 and Figure 3A,B).

Figure 2 shows the baseline parameters analyzed in Table 1 which decreased statistically significantly during the day shift.

Figure 3 shows the baseline parameters analyzed in Table 1 that increased statistically significantly during the night shift.

The onset-to-ED door time, the door-to-physician time for both emergency medicine and neurology physicians, the door-to-blood test time, the door-to-CT time, the door-to-CT results, and the door-to-needle time were calculated for the two groups (the ‘‘day shift’’, and ‘‘night shift’’ groups). The door-to-needle time was similar in all groups (*p* = 0.533). The door-to-CT and door-to-CT results were 19 and 39 min for the day shift, respectively, and 21 and 40 min for the night shift. The Shapiro–Wilk test confirmed normal distribution in all groups, and the unpaired t-test revealed statistically significant differences between the two groups (*p*= 0.037 and *p* = 0.02, respectively). The arrival mode in ED, with EMS or arrival via walk-in, was similar between the two ED shifts. (Table 2).

**Figure 4:**
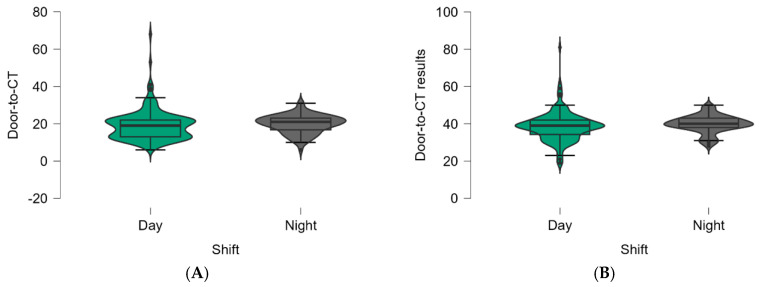
(**A**). Violin plot of the values of the door-to-CT time (minutes) between the two groups of patients (*p* = 0.037). (**B**) Violin plot of the values of the door-to-CT results time (minutes) between the two groups of patients (*p* = 0.02). The box plots inside the violins represent the median and interquartile ranges.

The door-to-CT and door-to-CT results times were increased statistically significantly during the night shift. (Figure 4A,B).

### 3.2. Analysis of Administration of Intravenous rt-PA during Day Shift or Night Shift

Three hundred forty-five patients with code stroke alert activations were recorded between 2020 and 2023, with a total of forty-six patients receiving IV thrombolysis. We also noted that in our study, the proportion of patients receiving thrombolysis was significantly higher during the night shift (*p* = 0.044) (Table 3).

### 3.3. The Analysis of ED Times Targets 

A logistic regression analysis considering the door-to-needle time (minutes) as the dependent variable demonstrated that the onset-to-ED time (*p* < 0.001) and door-to-physician (emergency medicine physicians) time (*p* = 0.021) are predictors for performing thrombolysis; the shorter the times for these two variables are, the more significantly increased the chance of thrombolysis is. (Table 4).

**Figure 5:**
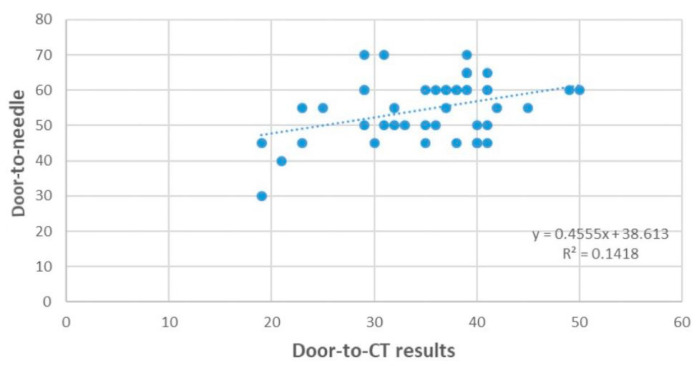
Linear regression plot.

Moreover, the dependence of the door-to-needle time (minutes) on the door-to-CT results (minutes) is significant, direct, and weak (*p* = 0.010, r = 0.377). (Figure 5).

## 4. Discussion

Evidence from previous studies indicates a direct relationship between the importance of stroke management times and the thrombolysis rate. We can state that patients with ischemic stroke in the ED are a priority, and compliance with these ED target times is directly correlated with their outcome and the thrombolysis rate [12,13,14]. Our study observed statistically significant differences in the ED time targets for code stroke alert management times between patients admitted during two ED shifts, a day shift (n = 178) and a night shift (n = 80). Our results show that admission to the emergency department during the night shift resulted in longer management times for door-to-CT and door-to-CT results. However, there was no delay in treatment delivery, with the proportion of patients receiving thrombolysis significantly higher during the night shift (*p* = 0.044). 

Despite the stroke protocol remaining consistent throughout the day, our data revealed a notable increase in door-to-CT and door-to-CT results during night shift hours. There are several potential explanations for this finding. Firstly, the fatigue experienced by the medical staff, including the 24 h on-call neurologist, can lead to longer reaction times when responding to code stroke alerts. Additionally, the reduced number of senior radiology residents available during night shifts means that all medical personnel have a more significant workload, which can contribute to delays in the overall process. Due to legislation in our country, it is only possible to preregister patients after they arrive in our jurisdiction. This legislation prohibits the transmission of patient-identifiable information by EMS while en route. Another aspect is that our study also covers the COVID-19 period. In recent years, the DTN time has improved significantly in Romania; however, the COVID-19 pandemic has affected this time measure. In 2017, the average of this time was 67 min, which decreased to 43 min in 2020. Yet, in 2023, it increased again to 60 min. The declaration of a state of emergency in Romania on 16 March 2020 did not initially affect the DTN time recorded in our national register for that month. As a result, the challenges faced by the healthcare system, including EMS and ED staff, over 30 months of disrupted processes and exhausted medical staff led to longer door-to-needle times. Our national stroke data analysis reflects this [9]. The improved time during the COVID-19 pandemic may be because physicians treated AIS as the same emergency as before or because during that period, the number of patients presenting for all diseases in the ED decreased after government restrictions, impacting first the door-to-physician (ED doctors) time. During that time, access and assessments of AIS patients with COVID-19 in the ED followed the same protocol; the only factor that could interfere with prolonged ED time targets was the requirement for physicians to wear mandatory personal protective equipment when monitoring and treating these patients.

Additionally, the health system protocol in our country requires confirmation from the patient or a witness before registration [5]. Unfortunately, this poses a challenge for stroke patients who may not be able to identify themselves due to aphasia or impaired consciousness. As a result, there is often a delay of 10 to 15 min as physicians search for a witness to identify the patient. CT imaging cannot be ordered as the patient must still be registered in the electronic entry system. We have been informed that many other hospitals experience similar delays in which imaging can only be ordered once the patient is electronically registered (personal communications). 

Ganti et al. conducted a study with a similar design to ours, also aiming to identify what factors in the emergency department impact the door-to-needle time in acute stroke patients eligible for intravenous thrombolysis and classifying their cohort as either “day shift” (6 a.m.–6 p.m.) or “night shift” (6 p.m.–6 a.m.) patients. Nearly half of the cohort, 49%, arrived during the daytime; 24% presented during the night shift, and the remaining 27% presented on the weekend. The majority, 85%, were brought to the hospital by EMS, while 15% of patients walked in. When examining the median DTN time, it was found that during the day shift, it took approximately 37 min (with an interquartile range of 26–51 and a range of 10–117). On the other hand, during the night shift, the median DTN time increased to 59 min (with an interquartile range of 39–89 and a range of 34–195). Interestingly, when a dedicated stroke team was present, the median DTN time significantly decreased to 36 min compared to 51 min when no one was available. The median door-to-CT time was also 24 min (with an interquartile range of 18–31 min). Univariate analyses revealed that arriving during the night shift (*p* < 0.0001), arriving as a walk-in (*p* = 0.0080), and experiencing a longer time to CT scan (*p* < 0.0001) were all associated with a longer DTN time. Conversely, the presence of a dedicated stroke team was associated with a significantly shorter DTN time (*p* < 0.0001) [15]. 

Within this examination of consecutive patients experiencing acute ischemic stroke and admitted within 4.5 h of symptom onset, a total of 258 individuals were eligible for thrombolysis. Astonishingly, only 17.83% (n = 46) of these patients received intravenous thrombolysis. This highlights the global underutilization of rt-PA administration, as recent international studies estimate that only 10–20% of eligible patients receive this treatment [16]. It is worth noting that Romania, according to the PA-CVA document, reports a mean national rt-PA administration rate lower than 10% (approximately 8.7%), making our study’s thrombolysis rate comparatively higher [17,18]. Similarly, China reports an alarmingly low national thrombolysis rate of only 2.4%, with intravenous rt-PA usage at a mere 1.6% [19].

Among the factors associated with the lack of performing thrombolysis in our study, no statistically significant correlation was found between the two shifts regarding the mode of arrival at the ED, whether it was via EMS (*p* = 0.894) or walk-in (*p* = 878), or risk factors such as the presence of high blood glucose (*p* = 0.925) or low platelets (*p* = 0.519). 

This lack of correlation is consistent with the literature, which reports different distributions of arrival modes and risk factors between the groups. For example, previous studies have shown a significant association between arriving during the night shift (*p* < 0.0001) and arriving without the emergency medical system (*p* = 0.0080) [20]. In a different study, about 18.1% of patients reported arriving at the ED by ambulance, while most arrived in private cars [21]. We consider the fact that there is no statistically significant difference in arrival mode types between our day and night shifts a strength of this study as it allows us to better analyze the impact of ED time targets on the administration of IV thrombolysis and which ED time is a predictor for an increased DTN time. 

Therefore, our data show that for day shifts, medians increased, for an onset-to-ED door time of 310 min (IQR, 190–340 min), a door-to-physician (emergency medicine doctor) time of 5 min (IQR, 3–9 min), and a door-to-physician (neurologist) time of 7 min (IQR, 5–10 min). For the night shift, the medians increased for a door-to-CT time of 21 min (IQR, 16.75–23 min), a door-to-CT results time of 40 min (IQR, 38–43 min), and a DTN time of 57.5 min (IQR, 46.25–60 min). Our study’s mean door-to-CT time of 21 min is under the recommended target of ≤25 min. A much-increased value of door-to-CT time (49.4 min) was registered in a study from Lebanon [21]. 

A comprehensive study was conducted over three years on patients diagnosed with acute stroke in the emergency departments of three Victorian hospitals, and it was found that out of all patients included, 71% (n = 2509) arrived by ambulance, while the remaining 29% (n = 1039) used private transport. Several factors were found to be significantly associated with ambulance arrival, including older age (*p* < 0.001), being born in Australia (*p* < 0.001), and speaking English at home (*p* = 0.003). The study also revealed that arriving by ambulance was independently linked to receiving prompt stroke care in the emergency department, coming within 2 h from symptom onset, being treated at an advanced stroke service center that offers thrombolysis, being triaged specifically for stroke, undergoing medical assessment within 25 min, and being referred for a CT scan within 45 min [22]. 

A study based on a project called HASTE (Hurry Acute Stroke Treatment and Evaluation) consisting of three study phases evaluated the effectiveness of four specific strategies in reducing the DTN time for intravenous alteplase in acute ischemic stroke patients. Employing a prospective pre- and post-intervention design, the study accounted for each strategy, secular trends, and patient characteristics. Notably, significant improvements in door-to-needle times were observed throughout the different phases of HASTE. Phase I involved a year-long data collection period and an analysis to assess the existing performance of the DTN. In Phase II, which spanned two years, a triage system was implemented to prioritize severe stroke cases within 4.5 h of onset. This approach aimed to concentrate the efforts of the stroke team on patients with the highest likelihood of requiring alteplase despite the high volume of milder or subacute stroke assessments encountered in their busy ED (with over 1500 stroke service admissions annually). During the HASTE phase III study, a new protocol was introduced to streamline the process for patients with a code stroke alert. Instead of being transferred to an ED bed, these patients were taken directly from the EMS stretcher to the CT scanner. Upon arrival at the emergency department, ED physicians swiftly evaluated the patient’s medical stability and neurological signs before promptly transporting the patient to the scanner on the same stretcher. After implementing these strategies, they observed significant enhancements in the DTN time. The administration of alteplase in the diagnostic imaging area led to a remarkable 32% reduction in the DTN time. Similarly, transferring the patient to the diagnostic imaging area using Emergency Medical Services stretcher resulted in a substantial 30% decrease in the DTN time. Registering the patient as unknown before their complete identification by family members or an informant yielded a 12% reduction in the DTN time. Additionally, employing simultaneous notification through a group pager for incoming patients with high stroke severity contributed to a statistically significant decrease in the DTN time of 11% [23]. 

In our analysis, we conducted a logistic regression to examine the impact of different ED times on the DTN time. We found that the onset-to-ED (*p* < 0.001) and door-to-physician (emergency medicine physician) times were significant predictors for the administration of thrombolysis. Specifically, we observed that shorter times for these two variables were associated with a significantly higher likelihood of receiving thrombolysis, as shown in Table 4. Recent research has indicated a correlation between decreased healthcare professionals and longer door-to-physician and DTN times [24]. Encouragingly, our findings revealed that the distribution of the capacity limitations for stroke patients arriving at the emergency department mirrored what happened in emergency shifts overall. We believe the factors contributing to the low rate of intravenous thrombolysis in our study are the following: delays during the triage and initial assessment of stroke patients in the emergency department, as can be observed in the results of our study. 

Furthermore, stroke symptoms can sometimes be subtle or mimic other conditions, and this can be worsened when the initial assessment is conducted by a resident rather than an ED specialist, leading to a delay in stroke identification and treatment, as we can note from our observations. It may be beneficial to conduct further investigations to gain a deeper understanding of how staffing models and the timing of patient arrival in the ED contribute to delays in stroke care, particularly during periods of increased ED crowding. Additionally, exploring the reasons behind neurologists’ decisions to not perform IV thrombolysis in eligible patients admitted to the ED during the time window could provide valuable insights, considering our research primarily focuses on ED management and time targets. 

The findings of this study have significant implications for medical practice, specifically in raising awareness about the importance of timely intervention for AIS patients through stroke code alerts. By analyzing ED time targets, this study has the potential to enhance acute stroke management in countries like Romania, where stroke incidence and mortality rates are alarmingly high. By identifying areas for improvement, we can optimize emergency department protocols and make a positive impact on stroke care not only nationally but also internationally. It is worth noting that this study examined the time window for AIS treatment and identified weaknesses that can be addressed to improve the thrombolysis procedure during emergency department management. So, we believe that the implementation of a process to bring the patient directly to the CT scanner from the emergency medical services stretcher or to start IV thrombolysis at the CT scanner are changes that can be implemented to shorten door-to-needle times and thereby increase reperfusion therapy rates as well as patient outcomes.

## 5. Study Limitations

It is essential to acknowledge the limitations of this study. While we conducted a retrospective analysis and obtained all timing information from patient medical records, there is still a possibility of unmeasured factors influencing our estimates. Despite our efforts to control for known variables associated with the DTN time, other potentially confounding variables may exist. We did not specifically investigate individual patient reasons for outliers, such as cases in which the stroke initially appeared mild but later worsened. However, a previous study conducted by our team, which focused on an emergency department located 3.5 km away from a thrombolysis hospital with a neurology department, did not find any significant statistical differences regarding ED time targets between patients who received thrombolysis and those who did not [12].

Further research will be required to address the predictors for the DTN time and understand the generalizability of our study’s results. This study was conducted in a high-volume academic center, and we anticipate that future studies will need to be carried out in different centers with varying acute stroke protocols. It is crucial to investigate why specific interventions were not implemented by documenting the reasons behind their omission. Although we did not collect data on stroke mimics or risk factors related to performed IV thrombolysis or a lack of IV thrombolysis, previous research suggests that only reducing the DTN time does lower the risk of complications [25]. It is important to note that specific changes may vary depending on hospital policies and structures. However, the impact of meeting or shortening these time targets can be assessed in other hospitals to determine their effect on the DTN time. Unfortunately, we could not analyze whether the same level of attention was consistently given to patients with code stroke alerts during different shifts or if they burdened the stroke team and CT or ED resources excessively. Nevertheless, it is crucial to assess the capacity of stroke programs in other centers based on their available resources. However, it is essential to remember that we did not include in this study patients in whom mechanical thrombectomy could probably have been performed after exceeding the 4.5 h target for IV thrombolysis [5] or patients with wake-up stroke due to the impossibility of performing a neuroimaging assessment using advanced techniques such as CT perfusion (CTP), which is available in only one hospital in Romania, or magnetic resonance imaging (MRI), which is not available for acute stroke.

## 6. Conclusions

This study identified higher door-to-CT and door-to-emergency medicine physician times associated with an increased DTN time. Further, door-to-CT and door-to-CT results times showed statistically significant increases during the night shift. These findings need to be considered when conducting quality improvements of hospital acute stroke protocols as they represent factors that can be addressed operationally.

## Figures and Tables

**Table 1:** Characteristics of analyzed group according to day shift (n = 178) and night shift (n = 80).

Variable	Shift	Valid	Mean ± SD	Median (IQR)	*p*
Physical characteristics					
Age, years	Day	178	67.29 ± 11.8	68 (61–75)	0.054
	Night	80	69.83 ± 12.68	71 (64–79)	
Height, cm	Day	178	171.52 ± 8.3	170 (165–178)	0.004 *
	Night	80	168.35 ± 7.35	168 (164.5–173.25)	
Weight, kg	Day	178	77.49 ± 12.45	79 (70–85)	0.157
	Night	80	75.15 ± 11.85	75 (68.75–81)	
SBP, mmHg	Day	178	156.56 ± 21.01	160 (140–170)	0.072
	Night	80	150.88 ± 19.44	150 (135–166.25)	
DBP, mmHg	Day	178	82.19 ± 13.13	80 (75–90)	0.166
	Night	80	79.69 ± 12.46	80 (70–90)	
GCS	Day	178	13.16 ± 3.36	15 (12–15)	0.269
	Night	80	12.61 ± 3.9	15 (12–15)	
NIHSS at presentation	Day	178	14.21 ± 5.34	14 (10–18)	0.331
	Night	80	14.8 ± 5.78	15 (10.75–19)	
Blood test sample results					
Platelets count, ×10^9^ μL	Day	178	224.19 ± 70.84	220 (175–254.75)	0.519
	Night	80	217.15 ± 64.61	218 (170.75–252)	
Hemoglobin, mg/dL	Day	178	13.74 ± 1.65	14 (13–15)	<0.001 *
	Night	80	12.75 ± 1.99	13 (12–14)	
Blood Glucose, mg/dL	Day	178	138.72 ± 48.25	125.5 (104–163.75)	0.925
	Night	80	138.43 ± 43.78	123 (105–175.25)	
Total Cholesterol, mg/dL	Day	178	188.93 ± 44.66	187.5 (162.5–220)	0.705
	Night	80	191.33 ± 51.35	189.5 (148.75–230.25)	
INR	Day	178	1.53 ± 1.41	1.14 (1.03–1.39)	0.039 *
	Night	80	1.79 ± 1.87	1.25 (1.1–1.44)	
Partial thromboplastin time, s	Day	178	29.62 ± 15.72	26.05 (23.5–30.2)	0.895
	Night	80	28.07 ± 9.23	26.3 (23.98–29.2)	
Prothrombin time, s	Day	178	16.11 ± 15.98	12.9 (12–14.38)	<0.001 *
	Night	80	19.38 ± 21.42	14.15 (12.8–15.55)	

* significant difference. SBP, systolic blood pressure; DBP, diastolic blood pressure; GCS, Coma Glasgow Score; INR, international normalized ratio. Values are expressed as means ± standard deviation (SD), by median (interquartile range), or by number (%).

**Table 2:** Time targets and arrival mode in the emergency department according to day shift (n = 178) and night shift (n = 80).

Variable	Shift	Valid	Mean ± SD	Median (IQR)	*p*
ED Time Targets (minutes)					
Onset-to-ED door time	Day	178	274.27 ± 101.33	310 (190–340)	0.082
	Night	80	248.25 ± 115.16	300 (127.5–330)	
Door-to-physician time (ED doctor)	Day	178	6.58 ± 5.12	5 (3–9)	0.232
	Night	80	5.453 ± 3.33	5 (3–8)	
Door-to-physician time(neurologist)	Day	178	8.15 ± 5.18	7 (5–10)	0.468
	Night	80	7.21 ± 3.02	7 (5–9)	
Door-to-blood samples	Day	178	8.06 ± 2.44	10 (5–10)	0.411
	Night	80	7.81 ± 2.62	10 (5–10)	
Door-to-CT	Day	178	19.12 ± 8.07	19 (13–22)	0.037 *
	Night	80	19.85 ± 4.84	21 (16.75–23)	
Door-to-CT results	Day	178	38.52 ± 7.76	39 (34.25–42)	0.02 *
	Night	80	39.84 ± 4.67	40 (38–43)	
Door-to-needle time	Day	26	55.19 ± 10.15	57.5 (46.25–60)	0.533
	Night	20	54 ± 6.41	55 (50–60)	
Arrival Mode					
Emergency medical services (EMS)	Day	178	(91) 51.12%		0.894
	Night	80	(41) 51.25%		
Arrival via walk-in	Day	178	(87) 48.87%		0.878
	Night	80	(39) 48.75%		

* significant difference.

**Table 3:** Analysis of IV thrombolysis administration between emergency department across day and night shifts.

		Thrombolysis		
Shift		No	Yes	Total
Day	Count	152	26	178
	% within row	85.393%	14.607%	100.000%
Night	Count	60	20	80
	% within row	75.000%	25.000%	100.000%
Total	Count	212	46	258
	% within row	82.171%	17.829%	100.000%
*p* = 0.044				

**Table 4:** Logistic regression (using the enter method) considers door-to-needle time as a dependent variable.

Variables in the Equation	B	S.E.	Wald	df	Sig.	Exp(B)	95% C.I.for EXP(B)	
							Lower	Upper
Onset-to-ED door (minutes)	−0.031	0.005	30.942	1	<0.001 *	0.97	0.959	0.98
Door-to-physician (ED doctor) (minutes)	−0.422	0.183	5.328	1	0.021 *	0.655	0.458	0.938
Door-to-neurologist (minutes)	0.289	0.193	2.258	1	0.133	1.336	0.916	1.948
Door-to-blood-samples (minutes)	−0.015	0.118	0.015	1	0.902	0.986	0.781	1.243
Door-to-CT (minutes)	−0.102	0.067	2.306	1	0.129	0.903	0.793	1.03
Door-to-CT results (minutes)	−0.081	0.058	1.901	1	0.168	0.923	0.823	1.035
Constant	9.235	2.225	17.235	1	<0.001	10,250.746		

* significant association; Cox and Snell R-Square = 0.44.

## Data Availability

The datasets are not publicly available, but de-identified data may be provided upon request from Popa Daian.

## References

[1] Wafa H.A., Wolfe C.D.A., Emmett E., Roth G.A., Johnson C.O., Wang Y. (2020). Burden of Stroke in Europe: Thirty-Year Projections of Incidence, Prevalence, Deaths, and Disability-Adjusted Life Years. Stroke.

[2] Di Cesare M., Perel P., Taylor S., Kabudula C., Bixby H., Gaziano T.A., McGhie D.V., Mwangi J., Pervan B., Narula J. (2024). The Heart of the World. Glob. Heart.

[3] Feigin V.L., Stark B.A., Johnson C.O., Roth G.A., Bisignano C., Abady G.G., Abbasifard M., Abbasi-Kangevari M., Abd-Allah F., Abedi V. (2021). Global, regional, and national burden of stroke and its risk factors, 1990–2019: A systematic analysis for the Global Burden of Disease Study 2019. Lancet Neurol..

[4] Stroke Alliance for Europe The Burden of Stroke in Europe—Challenges for Policy Makers. https://www.stroke.org.uk/sites/default/files/the_burden_of_stroke_in_europe_-_challenges_for_policy_makers.pdf.

[5] Priority Action for Interventional Treatment of Patients with Acute Stroke. Standard Operating Procedure Regarding the Patient Track and Therapeutic Protocol in Romania. https://legislatie.just.ro/Public/DetaliiDocument/209994.

[6] Manners J., Khandker N., Barron A., Aziz Y., Desai S.M., Morrow B., Delfyett W.T., Martin-Gill C., Shutter L., Jovin T.G. (2019). An interdisciplinary approach to inhospital stroke improves stroke detection and treatment time. J. NeuroInterventional Surg..

[7] Maestroni A., Mandelli C., Manganaro D., Zecca B., Rossi P., Monzani V., Torgano G. (2008). Factors influencing delay in presentation for acute stroke in an emergency department in Milan, Italy. Emerg. Med. J..

[8] Terecoasă E.O., Radu R.A., Negrilă A., Enache I., Cășaru B., Tiu C. (2022). Pre-Hospital Delay in Acute Ischemic Stroke Care: Current Findings and Future Perspectives in a Tertiary Stroke Center from Romania—A Cross-Sectional Study. Medicina.

[9] Tiu C., Terecoasă E.O., Tuță S., Bălașa R., Simu M., Sabău M., Stan A., Radu R.A., Tiu V., Cășaru B. (2023). Quality of acute stroke care in Romania: Achievements and gaps between 2017 and 2022. Eur Stroke J.

[10] Aho K., Harmsen P., Hatano S., Marquardsen J., Smirnov V.E., Strasser T. (1980). Cerebrovascular disease in the community: Results of a WHO collaborative study. Bull. World Health Organ..

[11] Goldstein L.B., Bertels C., Davis J.N. (1989). Interrater reliability of the NIH stroke scale. Arch. Neurol..

[12] Popa D., Iancu A., Petrica A., Buleu F., Williams C.G., Sutoi D., Trebuian C., Tudor A., Mederle O.A. (2024). Emergency Department Time Targets for Interhospital Transfer of Patients with Acute Ischemic Stroke. J. Pers. Med..

[13] Jones S., Moulton C., Swift S., Molyneux P., Black S., Mason N., Oakley R., Mann C. (2022). Association between delays to patient admission from the emergency department and all-cause 30-day mortality. Emerg. Med. J..

[14] Jones E.M., Boehme A.K., Aysenne A., Chang T., Albright K.C., Burns C., Beasley T.M., Martin-Schild S. (2015). Prolonged emergency department length of stay as a predictor of adverse outcomes in patients with intracranial hemorrhage. J. Crit. Care Med..

[15] Ganti L., Mirajkar A., Banerjee P., Stead T., Hanna A., Tsau J., Khan M., Garg A. (2023). Impact of emergency department arrival time on door-to-needle time in patients with acute stroke. Front. Neurol..

[16] De Souza A.C., Sebastian I.A., Zaidi W.A.W., Nasreldein A., Bazadona D., Amaya P., Elkady A., Gebrewold M.A., Vorasayan P., Yeghiazaryan N. (2022). Regional and national differences in stroke thrombolysis use and disparities in pricing, treatment availability, and coverage. Int. J. Stroke.

[17] Uivarosan D., Bungau S., Tit D.M., Moisa C., Fratila O., Rus M., Bratu O.G., Diaconu C.C., Pantis C. (2020). Financial burden of stroke reflected in a pilot center for the implementation of thrombolysis. Medicina.

[18] Sabau M., Bungau S., Buhas C.L., Carp G., Daina L.G., Judea-Pusta C.T., Buhas B.A., Jurca C.M., Daina C.M., Tit D.M. (2019). Legal medicine implications in fibrinolytic therapy of acute ischemic stroke. BMC Med. Ethics.

[19] Dong Q., Dong Y., Liu L., Xu A., Zhang Y., Zheng H., Wang Y. (2017). The Chinese Stroke Association scientific statement: Intravenous thrombolysis in acute ischaemic stroke. Stroke Vasc. Neurol..

[20] Dimitriou P., Tziomalos K., Christou K., Kostaki S., Angelopoulou S.M., Papagianni M., Ztriva E., Chatzopoulos G., Savopoulos C., Hatzitolios A.I. (2019). Factors associated with delayed presentation at the emergency department in patients with acute ischemic stroke. Brain Inj..

[21] El Sayed M.J., El Zahran T., Tamim H. (2014). Acute stroke care and thrombolytic therapy use in a tertiary care center in Lebanon. Emerg. Med. Int..

[22] Kinsella D., Mosley I., Braitberg G. (2018). A Retrospective Study Investigating: Factors associated with mode of arrival and emergency department management for patients with acute stroke. Australas. Emerg. Care.

[23] Kamal N., Holodinsky J.K., Stephenson C., Kashayp D., Demchuk A.M., Hill M.D., Vilneff R.L., Bugbee E., Zerna C., Newcommon N. (2017). Improving Door-to-Needle Times for Acute Ischemic Stroke: Effect of Rapid Patient Registration, Moving Directly to Computed Tomography, and Giving Alteplase at the Computed Tomography Scanner. Circ. Cardiovasc. Qual. Outcomes.

[24] Jaffe T.A., Goldstein J.N., Yun B.J., Etherton M., Leslie-Mazwi T., Schwamm L.H., Zachrison K.S. (2020). Impact of Emergency Department Crowding on Delays in Acute Stroke Care. West. J. Emerg. Med..

[25] Man S., Solomon N., Grory B.M., Alhanti B., Uchino K., Saver J.L., Smith E.E., Xian Y., Bhatt D.L., Schwamm L.H. (2023). Shorter Door-to-Needle Times Are Associated With Better Outcomes After Intravenous Thrombolytic Therapy and Endovascular Thrombectomy for Acute Ischemic Stroke. Circulation.

